# Spatial metabolic heterogeneity of sugar–acid balance and pigment accumulation in distinct color regions of *Yanzhihong* apricot (*Prunus armeniaca* L.) revealed by MALDI-IMS

**DOI:** 10.3389/fpls.2025.1636734

**Published:** 2025-08-14

**Authors:** Yang Zhao, Yanhong He, Weiyuan Xing, Yu-e Bai, Hui Li, Dongmei Ye, Min Kang

**Affiliations:** Academy of Forestry, Inner Mongolia Agricultural University, Hohhot, China

**Keywords:** MALDI-IMS mass spectrometry imaging technology, metabolic spatial heterogeneity, sugar-acid metabolism, pigment distribution, fruit quality

## Abstract

This study is the first to apply spatial metabolomics techniques, MALDI-IMS to investigate differential distributions of sugars, organic acids, and pigments in the red and yellow regions of Yanzhihong apricot fruit. PCA and OPLS-DA analyses indicated that sucrose was significantly higher in the red region (21.39 mg/g) than in the yellow region (17.79 mg/g). In contrast, the yellow region exhibited significantly greater levels of fructose (31.54 mg/100g), ascorbic acid (11.03 mg/g), malic acid (3.61 mg/g), and citric acid (6.41 mg/g) than the red region (16.88mg/100g, 8.29, 2.34, and 4.27 mg/g, respectively). The red region also exhibited higher carotenoid levels (29.25 mg/g) and the anthocyanin cyanidin-3-O-(6’’-O-malonyl)glucoside, primarily in the peel and adjacent tissues, thereby enhancing pigment deposition and antioxidant capacity. These findings demonstrate notable spatial variability in sugar–acid metabolism and pigment accumulation between red and yellow fruit regions, which not only determine taste and color but also provide valuable insights for targeted fruit quality improvement and breeding of apricot cultivars with optimized sensory and nutritional traits.

## 1 Introduction

Apricot (*Prunus armeniaca* L.) is highly valued by both consumers and growers for its sweet flavor, vibrant peel color, and rich nutritional content (Crouzet et al., 1990; Jiang et al., 2019; Zhou et al., 2020). Sugars, organic acids, and pigments are key determinants of fruit quality exhibiting complex dynamic changes during fruit development and ripening (Karatas, 2022). However, the spatial distribution and dynamic changes of sugars, acids, and pigments within apricot fruit remain poorly understood, and the specific accumulation patterns of these metabolites in different fruit regions have not been fully elucidated.

The taste of apricot fruit is primarily determined by sugars and organic acids, with their balance being a key criterion for evaluating fruit flavor. Soluble sugars play a crucial role in fruit physiological development, nutrient synthesis, and the accumulation of flavor compounds. The main sugars in apricots include sucrose, fructose, glucose, and sorbitol (Tian et al., 2023), whereas the dominant organic acids are citric acid and malic acid. The predominance of malic acid results in a more acidic taste, whereas a higher citric acid content enhances palatability (Su et al., 2020; Mekhemar, 2021; Cui et al., 2022). In addition to serving as precursors for anthocyanin biosynthesis, sugars can also act as hormonal signal molecules in plants, playing regulatory roles similar to those of hormones (Hrazdina et al., 1984; Seyffert, 1987; Das et al., 2012). Studies have shown that sucrose can activate genes associated with anthocyanin synthesis, such as *MYB75/PAP1*, *CHS* (chalcone synthase), and *DFR* (dihydroflavonol 4-reductase), thereby promoting anthocyanin accumulation (Boss et al., 1996; Zhang et al., 2013; Li et al., 2014; Liu et al., 2024).

Fruit coloration in apricots is primarily determined by carotenoids and anthocyanins, both of which are critical for assessing fruit quality. Anthocyanins, a subclass of flavonoids widely distributed in nature, impart red, blue, and purple hues to flowers, fruits, leaves, seeds, and roots (Tanaka et al., 2008). Research indicates that the predominant anthocyanin in apricot fruit is cyanidin-3-O-rutinoside, which is significantly more abundant in red-colored fruits than in fruits of other colors (Huang et al., 2019). Additionally, secondary pigments, including cyanidin-3-O-glucoside, pelargonidin-3-O-glucoside, quercetin-rutinoside, and kaempferol-3-O-glucoside, also contribute to fruit color formation (Griesser et al., 2008; Fernandez-Moreno et al., 2016; Gao et al., 2020). Yellow and orange apricots owe their coloration to high carotenoid content, which not only varies in composition but also plays a crucial role in peel pigmentation (Kan and Bostan, 2010a). During fruit ripening, chlorophyll degradation, carotenoid synthesis, and anthocyanin accumulation collectively drive dynamic changes in peel coloration (Ruiz et al., 2005; Ayour et al., 2016). Certain flavonoid compounds also influence fruit flavor and pigmentation, such as 4β-(S-cysteinyl)-epicatechin/catechin, naringenin chalcone, and escin A/B (Agati et al., 2012; Bakir et al., 2020).

The rapid advancement of spatial metabolomics has provided a novel perspective for investigating the spatial distribution of fruit metabolites. By integrating high-resolution mass spectrometry with microscopic imaging, researchers can obtain spatial information on metabolite distribution at the cellular or tissue level, providing deeper insights into plant growth, development, and responses to environmental changes (Montini et al., 2020; Zhao et al., 2021). The spatial distribution of soluble sugars and organic acids is considered a key determinant of fruit flavor, as their ratio plays a crucial role in balancing sweetness and acidity (de Jesús Ornelas-Paz et al., 2013). For instance, in apples, sucrose is more concentrated in the outer fruit flesh, while sorbitol accumulates in the core, directly influencing fruit taste and sweetness distribution (Horikawa et al., 2019). In melons, studies have revealed significant differences in sugar and acid distribution across fruit developmental stages and tissues, with these spatial gradients closely linked to fruit flavor and nutritional enhancement (Moing et al., 2011). In kiwifruit, the pigment accumulation mechanism in red-fleshed varieties has been systematically characterized, revealing a highly coordinated regulatory network between sugar and acid metabolism (Mao et al., 2024). Additionally, the accumulation of anthocyanins and carotenoids has been shown to play a pivotal role in fruit coloration. In strawberries, anthocyanins are predominantly localized in the outer fruit tissues, contributing to the deep red color of mature fruit (Wang et al., 2021).

Currently, research on the spatial distribution of sugars, acids, and pigments in apricot fruit remains limited. Most existing studies focus on the overall metabolic profile of the fruit, overlooking the heterogeneity between different regions of the fruit. For the first time, this study applies spatial metabolomics to analyze the metabolic differences in distinct color regions of *Yanzhihong* apricot fruit. By employing spatial metabolomics techniques, this study aims to investigate the spatial distribution of sugars, acids, and pigments within apricot fruit, addressing a significant research gap in fruit metabolic localization.

## 2 Materials and methods

### 2.1 Plant materials

*Yanzhihong* apricot fruits were harvested from an orchard in Wanjiagou, Hohhot. To ensure the representativeness of the samples, a preliminary experiment was conducted with ten randomly selected mature apricot fruits. An *a priori* power analysis was performed using G*Power software (effect size *d* = 1.2, significance level *α* = 0.05, and sample size per group = 10), yielding a power value of 0.85. This result confirmed that the sample size provided sufficient statistical power (>0.8) to detect metabolic differences between groups. After harvest, a portion of the fruits was immediately dissected using a scalpel to obtain the target tissue regions for spatial metabolomics analysis, which were then placed on dry ice. The remaining fruits were cut into red and yellow sections, stored in centrifuge tubes on dry ice, and transported to the laboratory for physiological measurements.

### 2.2 Detection of physiological content

#### 2.2.1 Citrate content determination

Citrate is a product of the first reaction in the tricarboxylic acid cycle and participates in physiological metabolic activities such as respiration. Iron(III)-sulfosalicylic acid forms a purple-red complex, and citrate can reduce the color of this complex to orange-red. At a wavelength of 470 nm, the decrease in absorbance is proportional to the citrate content under certain conditions, allowing the determination of citrate content in the sample.

Tissue Sample Treatment: Weigh approximately 0.1 g of tissue, add 1 mL of extraction solution, and homogenize on ice. Centrifuge at 12,000 rpm, 4°C for 10 minutes. Add reagents as instructed in the manual for detection. All steps are performed strictly according to the manual.

#### 2.2.2 Sucrose-glucose-fructose content determination

Under the action of specific enzymes, sucrose is converted into glucose and fructose, and glucose, through the enzyme complex including hexokinase, reduces NADP+ to NADPH. By measuring the increase in NADPH at 340 nm, the contents of sucrose, glucose, and fructose can be determined separately.

Tissue Sample: Weigh approximately 0.1 g of tissue, add 1 mL of distilled water, and homogenize on ice. Centrifuge at 12,000 rpm at room temperature for 10 minutes. Add reagents as instructed in the manual for detection. All steps are performed strictly according to the manual.

#### 2.2.3 Reduced ascorbic acid content determination

Reduced ascorbic acid (AsA) can reduce ferric ions (Fe^3+^) to ferrous ions (Fe^2+^), and the ferrous ions react with red phenantroline to form a red complex. This complex has a characteristic absorption peak at 534 nm, and its intensity is directly proportional to the content of reduced ascorbic acid.

Tissue Sample Treatment: Weigh approximately 0.1 g of tissue, add 1 mL of pre-cooled extraction solution, and homogenize on ice. After extracting at room temperature for 10 minutes, centrifuge at 12,000 rpm, 4°C for 10 minutes. Add reagents as instructed in the manual for detection. All steps are performed strictly according to the manual.

#### 2.2.4 Total anthocyanin content determination

Anthocyanins are measured by the pH differential method, where the color of anthocyanins changes with pH, while the characteristic spectrum of interfering substances remains unaffected. At pH 1, anthocyanins appear red in the 2-phenylbenzopyran form; at pH 4.5, they are colorless in the methanol pseudobase form. The total amount is calculated based on the difference in absorbance at the two pH values (usually 1.0 and 4.5), where the largest and most stable absorbance difference occurs.

Tissue Sample Treatment: For hydrated samples, weigh approximately 0.5 g, add 1 mL of extraction solution, and homogenize. Extract at 75°C for 25 minutes with shaking. If any loss of extraction solution occurs, make up the volume to 1 mL. Centrifuge at 12,000 rpm for 10 minutes at room temperature. For solid dry samples, grind and sieve through a 40-mesh sieve. Weigh 0.02 g of the sieved dry sample, add 1 mL of extraction solution, and proceed with reagent addition as per the manual. All steps are performed strictly according to the manual.

#### 2.2.5 L-malic acid content determination

Malic acid is oxidized by malate dehydrogenase, and the resulting NADH reacts with a color reagent to form a colored substance. The amount of this colored substance formed at 450 nm can be used to calculate the malic acid content.

Tissue Sample Treatment: Weigh approximately 0.1 g of tissue, add 1 mL of extraction solution, and homogenize on ice. Transfer the crude extract into an EP tube and centrifuge at 12,000 rpm, 4°C for 10 minutes. Add reagents as instructed in the manual for detection. All steps are performed strictly according to the manual.

### 2.3 Fruit sectioning and sample preparation

Frozen sections of apricot tissue were prepared using a Leica CM1950 cryostat at a thickness of 50 μm. The fruit tissues samples, retrieved from a −80°C freezer, were equilibrated at -20°C in the cryostat for 1 hour before sectioning. The samples were mounted onto sample holders, and their orientation was carefully adjusted prior to securing them onto the cryostat’s positioning stage. The sections were transferred using a pre-cooled brush onto pre-chilled indium tin oxide (ITO) glass slides. To ensure adhesion, the back of the slides was pressed against the hand until the sections became transparent. Gentle finger rubbing on the back of the slide facilitated the evaporation of residual moisture, turning the sections from transparent to white. The prepared ITO slides were then dried in a vacuum desiccator for 30 minutes.

### 2.4 Matrix spraying

A 15 mg/mL solution of 2,5-dihydroxybenzoic acid (DHB) was prepared using a 90:10 acetonitrile:water solvent system. The DHB matrix solution was evenly sprayed onto the tissue sections using a TM-Sprayer matrix deposition system. The spraying parameters were set as follows: temperature, 60°C; flow rate, 0.1 mL/min; pressure, 7 psi; 25 spraying cycles, with a drying time of 5 seconds between cycles.

### 2.5 Mass spectrometry imaging

The matrix-coated ITO slides were placed on the mass spectrometer target plate(Mass Spectrometer Model: tims TOF flex, Mass Resolution: 40,000, Laser Scan Count per Pixel: 400, Laser Energy: 80%, Germany). The *DataImaging* software (Bruker) was used to define the tissue regions for analysis, with an imaging resolution of 50 μm (i.e., the smallest unit of the 2D matrix was 50 μm × 50 μm). The imaging area was divided into a two-dimensional array of sampling points according to the sample size. The detection range was set to 50–1300 Da. Under the same laser energy, the tissue sample is detected by focusing the laser beam onto the sample through an optical path and continuously scanning the sample. The released molecular ions were then detected by the mass spectrometer, providing mass-to-charge ratio (*m/z*) information and raw signal intensity data for each pixel. The raw data were imported into *SCiLS Lab* software, where they were normalized using the Root Mean Square (RMS) method. This process generated relative intensity values for different *m/z* signals at each spatial point, which were subsequently visualized as heatmap images (Deininger et al., 2011).

### 2.6 Data processing and statistical analysis

Standardized metabolic data were analyzed using multivariate statistical methods, including Principal Component Analysis (PCA) and Orthogonal Partial Least Squares Discriminant Analysis (OPLS-DA), to evaluate differences between fruit regions. One-way analysis of variance (ANOVA) was used to determine significant differences in metabolite concentrations between groups. Pearson correlation coefficients were calculated to assess linear relationships among metabolites, particularly between pigment accumulation and sugar–acid ratios. To control for multiple comparisons, false discovery rate (FDR) correction was applied to all p-values. Additionally, metabolic pathway enrichment analysis was performed using the Kyoto Encyclopedia of Genes and Genomes (KEGG) database to interpret the functional significance of metabolite variations.

## 3 Results

### 3.1 Physiological index analysis

The *Yanzhihong* apricot is distinguished by its unique appearance, characterized by two distinct color regions—red and yellow. These regions not only exhibit significant visual differences but also display distinct physicochemical properties. To systematically investigate the biochemical composition of these fruit regions, this study measured the contents of glucose, fructose, sucrose, ascorbic acid, malic acid, citric acid, chlorophyll *a*, chlorophyll *b*, carotenoids, and total anthocyanins.

**Figure 1:**
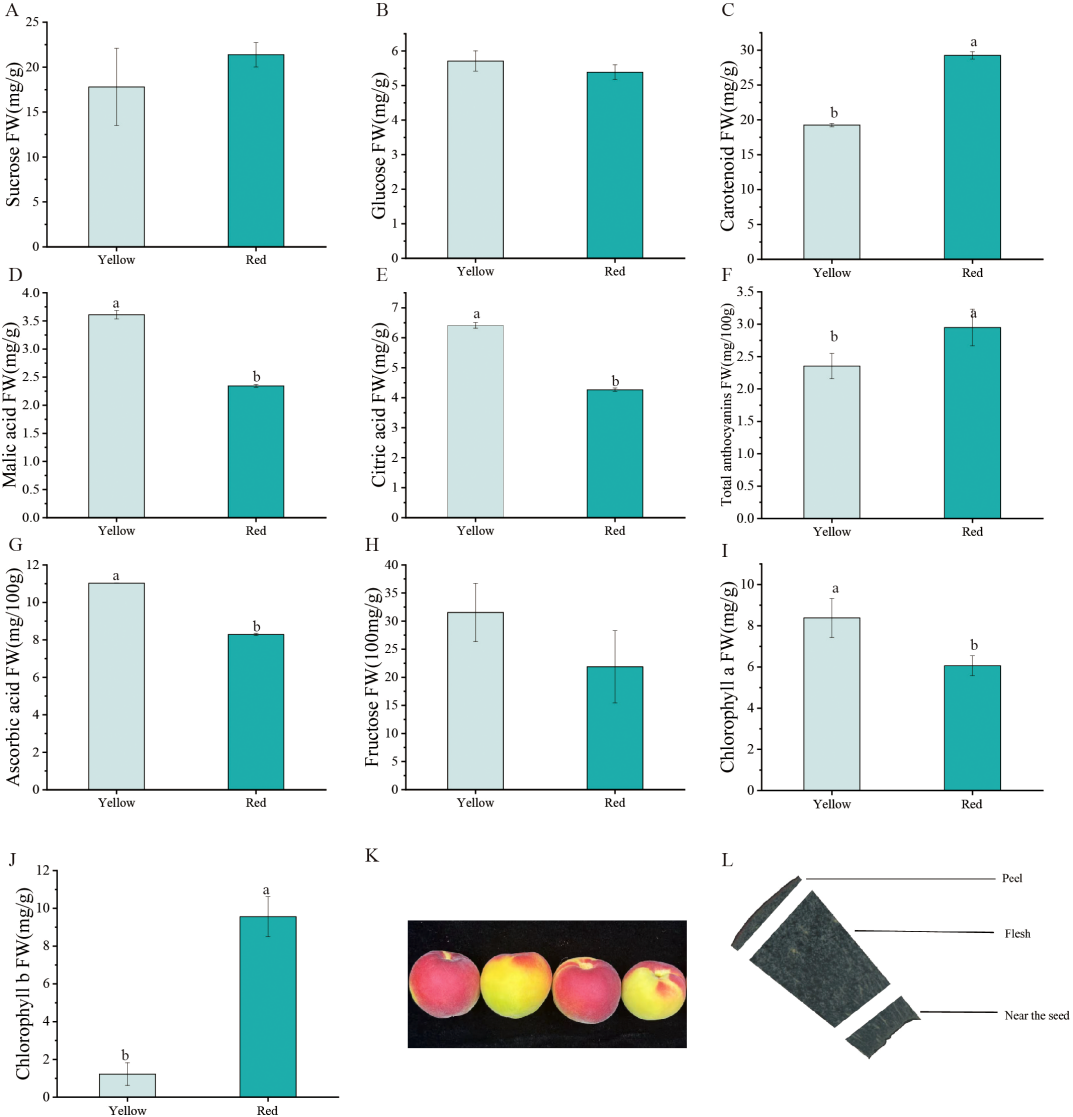
**(A-J)** Physiological content distribution in Yanzhihong apricot. **(K)** Color differentiation of Yanzhihong apricot fruit **(L)** Sectioning position of Yanzhihong apricot fruit. ‘a’ and ‘b’ letters indicate statistical differences (p < 0.05).

The results revealed differences in the relative abundance of various sugars between the two color regions (**Figure 1**). Glucose levels showed no significant difference between the yellow (5.71 mg/g) and red (5.39 mg/g) regions. However, fructose content was significantly higher in the yellow region (31.54 mg/100g) than in the red region (16.88 mg/g), whereas sucrose content was markedly higher in the red region (21.39 mg/g) compared to the yellow region (17.79 mg/g). This difference suggests that the yellow region primarily derives its sweetness from fructose, while the red region’s sweetness is more dependent on sucrose. The differential sugar distribution may be influenced by localized regulation of sugar metabolism pathways and enzyme activity. Significant differences were also observed in the distribution of organic acids. The yellow region exhibited higher levels of ascorbic acid (11.03 mg/100g), malic acid (3.61 mg/g), and citric acid (6.41 mg/g) compared to the red region (8.29 mg/100g, 2.34 mg/g, and 4.27 mg/g, respectively). These results indicate that the yellow region possesses a more pronounced acidic flavor profile.

Regarding pigment accumulation, chlorophyll *a* content was higher in the yellow region (8.38 mg/g) than in the red region (6.06 mg/g), whereas chlorophyll *b* content was substantially higher in the red region (9.56 mg/g) than in the yellow region (1.89 mg/g). Additionally, carotenoid content was significantly elevated in the red region (29.25 mg/g) compared to the yellow region (19.27 mg/g), contributing to the enhanced color saturation of the red region. The accumulation of anthocyanins further intensified the red hue of the fruit, albeit with a relatively low overall concentration. The red region exhibited a slightly higher anthocyanin content (2.95 mg/100g) than the yellow region (2.35 mg/100g), reinforcing its distinct color.

### 3.2 Relationship between sugars, organic acids, anthocyanins, and carotenoids in apricot fruit

This study systematically investigated the interrelationships among key metabolites, including sugars, organic acids, anthocyanins, and carotenoids, to elucidate their metabolic interactions (**Supplementary Figure S1**). The results demonstrated significant correlations between these metabolites, highlighting their complex regulatory relationships within metabolic pathways.

A particularly strong positive correlation was observed between ascorbic acid and citric acid (*r* = 0.9971). Conversely, ascorbic acid showed a significant negative correlation with Cyanidin-3-O-(6’’-O-malonyl) glucoside (*r* = -0.9795), suggesting that an increase in ascorbic acid may suppress the synthesis of certain anthocyanins, thereby influencing fruit coloration and pigmentation intensity. Furthermore, citric acid exhibited a strong negative correlation with carotenoid accumulation (*r* = -0.9953), indicating a potential inhibitory role of citric acid in carotenoid biosynthesis. Similarly, malic acid was also negatively correlated with carotenoid levels (*r* = -0.9916), further supporting the regulatory role of organic acids in carotenoid metabolism.

The metabolic relationship between anthocyanins and carotenoids was also noteworthy. Pelargonidin 3-O-beta-D-glucoside 5-O-(6-coumaroyl-beta-D-glucoside) exhibited a negative correlation with carotenoid accumulation (*r* = -0.9858), and a similar trend was observed for Cyanidin-3-O-(6’’-O-malonyl) glucoside (*r* = -0.9780). These findings suggest that the balance between anthocyanins and carotenoids may influence the final fruit coloration and the overall nutritional composition.

### 3.3 PCA and OPLS-DA analysis

**Figure 2:**
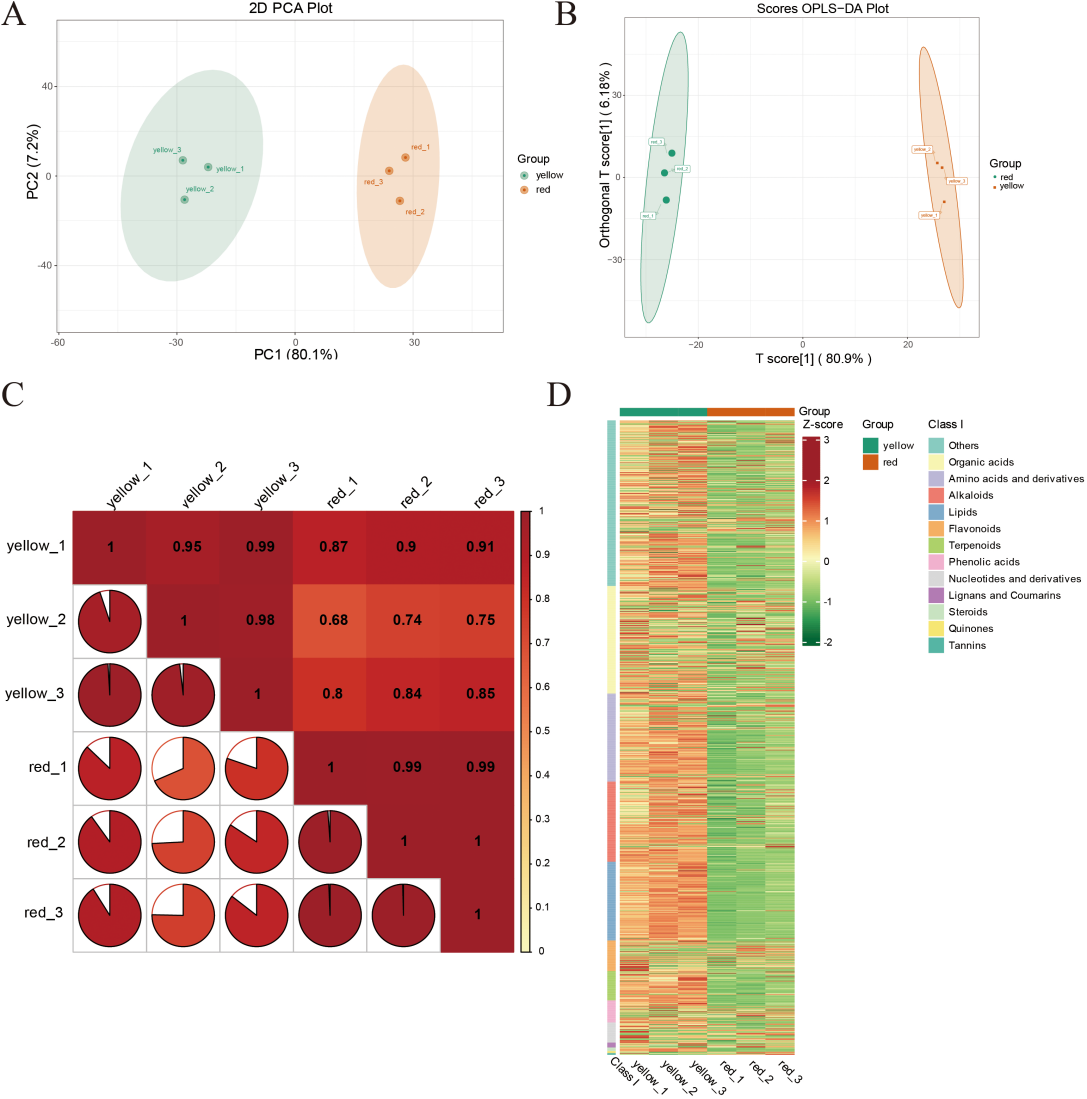
**(A)** Principal Component Analysis (PCA) plot showing PC1 (first principal component), PC2 (second principal component), and PC3 (third principal component). The percentages indicate the proportion of the dataset’s variance explained by each component. Each point in the plot represents a sample, with samples from the same group indicated by the same color. “Group” refers to the categorization of the samples. **(B)** The x-axis represents the predictive principal component, which highlights the differences between groups; the y-axis represents the orthogonal principal component, which shows variations within groups. The percentages indicate the explanation rate of each component for the dataset. **(C)** The x and y axes represent sample names, with colors changing from red to yellow indicating a decrease in correlation strength. The area of the pie segments in the plot represents the correlation coefficient size between the samples corresponding to the x and y axes. **(D)** The horizontal axis lists sample names, and the vertical axis lists metabolite information. “Group” refers to the categorization of the samples. Different colors represent different relative content levels normalized and are used to fill the grid, where red indicates higher content and green indicates lower content.

**Figure 2A** presents the results of Principal Component Analysis (PCA) of the differently colored regions (yellow and red) of apricot fruit, revealing the overall metabolic differences between the two groups. The x-axis represents the first principal component (PC1), which explains 80.1% of the variance, while the y-axis represents the second principal component (PC2), accounting for 7.2% of the variance. The PCA score plot clearly demonstrates a distinct separation between yellow and red fruit samples in the PC1–PC2 plane, indicating significant differences in metabolic characteristics between these color regions. The yellow fruit samples (*yellow_1*, *yellow_2*, *yellow_3*) clustered in the lower PC1 region, whereas the red fruit samples (*red_1*, *red_2*, *red_3*) were concentrated in the higher PC1 region. Furthermore, the high intra-group clustering suggests that the metabolic features associated with fruit color are highly stable and reproducible. **Figure 2B** displays the results of Orthogonal Partial Least Squares Discriminant Analysis (OPLS-DA), which further distinguishes the metabolic profiles of the yellow and red fruit samples. The x-axis represents the predictive principal component [T score (Zhou et al., 2020)], explaining 80.9% of the variance, while the y-axis represents the orthogonal principal component [Orthogonal T score (Zhou et al., 2020)], explaining 6.18% of the variance. The analysis revealed a clear separation between the yellow and red fruit samples along the T score (Zhou et al., 2020) axis, with yellow fruit samples (*yellow_1*, *yellow_2*, *yellow_3*) clustering on the right and red fruit samples (*red_1*, *red_2*, *red_3*) on the left. This distinct separation pattern further confirms systematic differences in the chemical composition and physicochemical properties of the two groups. Compared to PCA, OPLS-DA provided a more targeted classification by enhancing the visualization of inter-group differences while minimizing the influence of irrelevant variables, resulting in a clearer distinction between the sample groups. **Figure 2C** presents the correlation matrix of the different colored regions, providing insights into the overall consistency and variability in metabolite composition. The yellow fruit group (*yellow_1*, *yellow_2*, *yellow_3*) exhibited high internal correlation, with correlation coefficients approaching 1, indicating extremely stable metabolic composition with minimal inter-individual variation. In contrast, while the red fruit group (*red_1*, *red_2*, *red_3*) also showed high correlation, the values were slightly lower than those of the yellow group, suggesting a higher degree of metabolic variability within the red fruit samples. **Figure 2D** displays the heatmap of metabolite abundance in different fruit regions, categorizing metabolites into different classes, such as organic acids, amino acids and derivatives, and flavonoids. Standardized processing was applied to facilitate visualization of metabolite expression levels across samples. The relative abundance of metabolites in different samples further highlighted systematic metabolic differences between the yellow and red fruit regions. The yellow fruit exhibited higher expression levels in multiple metabolite categories, particularly in organic acids, flavonoids, and amino acids. In contrast, the red fruit showed lower or more dispersed metabolite expression in these categories, particularly for flavonoids and organic acids, which exhibited greater variability.

Overall, the heatmap analysis clearly illustrated the metabolic divergence between different fruit color regions. The yellow fruit exhibited a more stable metabolic profile with consistently higher expression levels across various metabolite categories, whereas the red fruit displayed greater variability, suggesting that its color formation, flavor profile, and nutritional value may be influenced by more complex regulatory mechanisms.

### 3.4 Differential metabolite analysis and metabolic pathway enrichment in red and yellow apricot fruit regions

**Figure 3:**
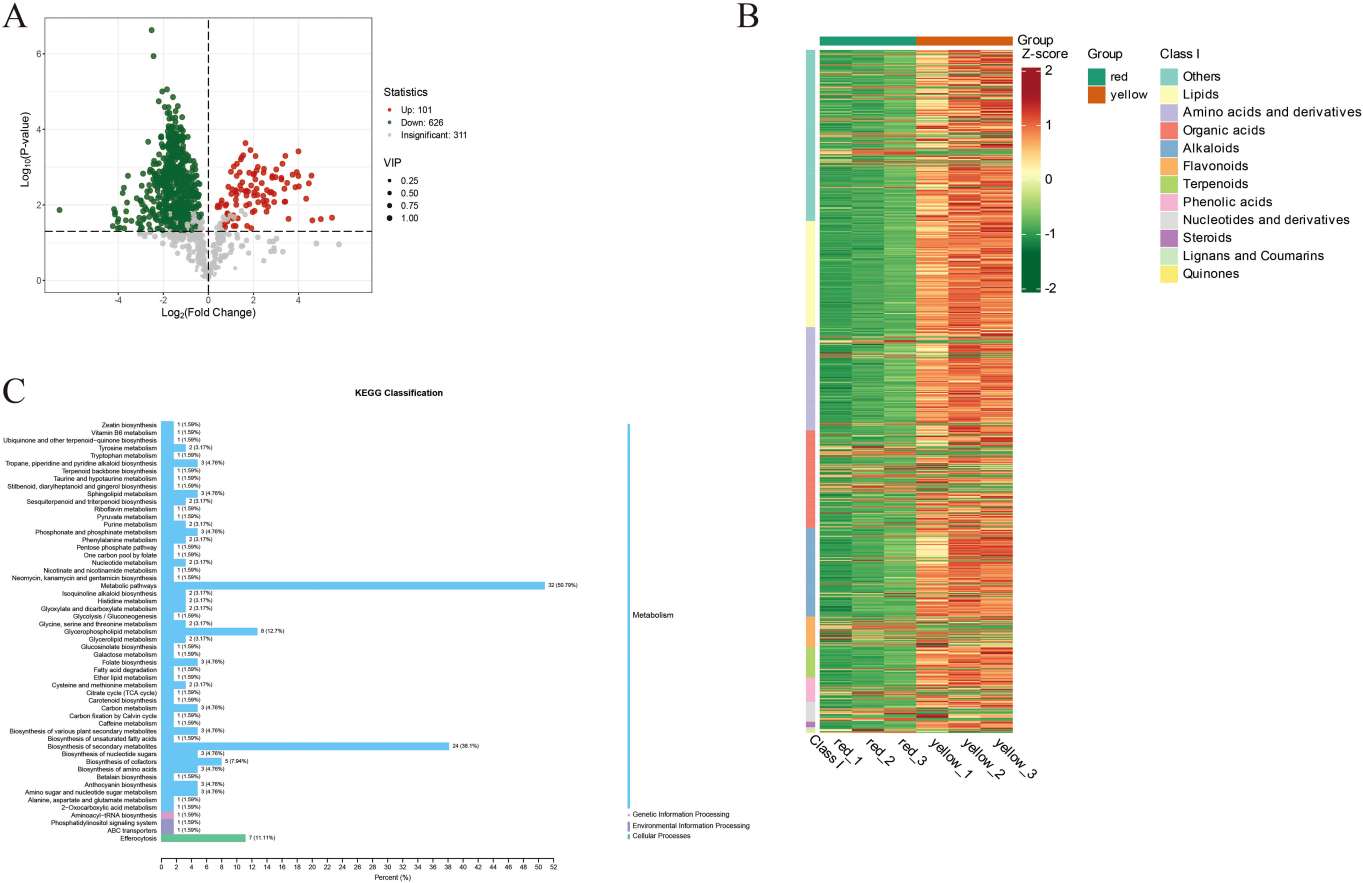
**(A)** The volcano plot where each point represents a metabolite. Green points indicate downregulated differential metabolites, red points indicate upregulated differential metabolites, and gray points represent metabolites that were detected but did not show significant differences. **(B)** The horizontal axis lists sample names, and the vertical axis lists differential metabolite information. “Group” refers to the categorization of the samples. Different colors represent different relative content levels that have been normalized and are used to fill the grid, where red indicates higher content and green indicates lower content. **(C)** The y-axis lists the names of KEGG metabolic pathways, and the x-axis shows the number of differential metabolites annotated in each pathway and their proportion relative to the total number of annotated differential metabolites.

**Figure 3A** presents the differential metabolite analysis between the red and yellow regions of apricot fruit. The volcano plot displays Log_2_(Fold Change) on the x-axis, representing the magnitude of metabolite expression changes, while the y-axis shows Log_10_(*P-value*), reflecting statistical significance. Each point represents a metabolite, with its color, size, and position indicating statistical significance, expression fold change, and Variable Importance in Projection (VIP) values, respectively. Overall, most metabolites (gray points) exhibited significant expression differences between the yellow and red fruit regions. However, distinct clusters of upregulated (red points) and downregulated (green points) metabolites highlighted the pronounced metabolic differences between the two color regions. Specifically, 101 metabolites were significantly upregulated in the red fruit, while 626 metabolites were significantly downregulated. Additionally, 311 metabolites showed no significant changes, suggesting stable expression levels across both fruit regions.

**Figure 3B** illustrates the enrichment patterns of various metabolite classes based on KEGG metabolic pathway analysis. The metabolites analyzed include lipids, amino acids and derivatives, organic acids, alkaloids, flavonoids, terpenoids, phenolic acids, nucleotides and derivatives, steroids, lignans and coumarins, and quinones, which display significant variations in abundance across different metabolic pathways. Heatmap analysis reveals marked differences in metabolite abundance, with red and yellow indicating high levels, while green signifies lower levels. These findings provide compelling evidence for the functional roles and regulation of metabolites across metabolic pathways, offering new insights into the dynamic changes within metabolic networks and their biological underpinnings.

**Figure 3C** further illustrates the results of Kyoto Encyclopedia of Genes and Genomes (KEGG) pathway enrichment analysis, revealing metabolic differences between the red and yellow regions of apricot fruit. The results indicate that primary metabolic pathways accounted for the highest proportion (50.79%), suggesting that most differential metabolites are involved in essential physiological processes such as carbohydrate metabolism and amino acid metabolism. These differences highlight the variations in fundamental metabolic activities between the two fruit regions.

The biosynthesis of secondary metabolites comprised 38.1% of the differential metabolic pathways. These compounds play a crucial role in plant defense mechanisms, antioxidant capacity, and aroma formation, potentially influencing fruit flavor and coloration. Amino acid metabolism accounted for 5.94%, suggesting that differences in amino acid synthesis and metabolism may contribute to variations in taste and nutritional composition.

Carbohydrate and glycogen metabolism pathways represented 3.17% of the enriched pathways, indicating that differences in sugar metabolism may regulate apricot sweetness and energy storage. Lipid metabolism accounted for 1.59%, suggesting potential effects on fruit ripening, antioxidant properties, and oil content. Nucleotide metabolism made up 2.13%, implying a role in cell division, gene expression, and fruit maturation regulation.

Furthermore, variations in the biosynthesis pathways of isoflavones and flavonoids highlighted their significant roles in pigment accumulation, flavor formation, and antioxidant properties in apricot fruit. These findings provide valuable insights into the metabolic mechanisms underlying color differentiation in apricot fruit and their potential implications for fruit quality and sensory attributes.

### 3.5 Spatial distribution of sugars, acids, and pigments

**Figure 4:**
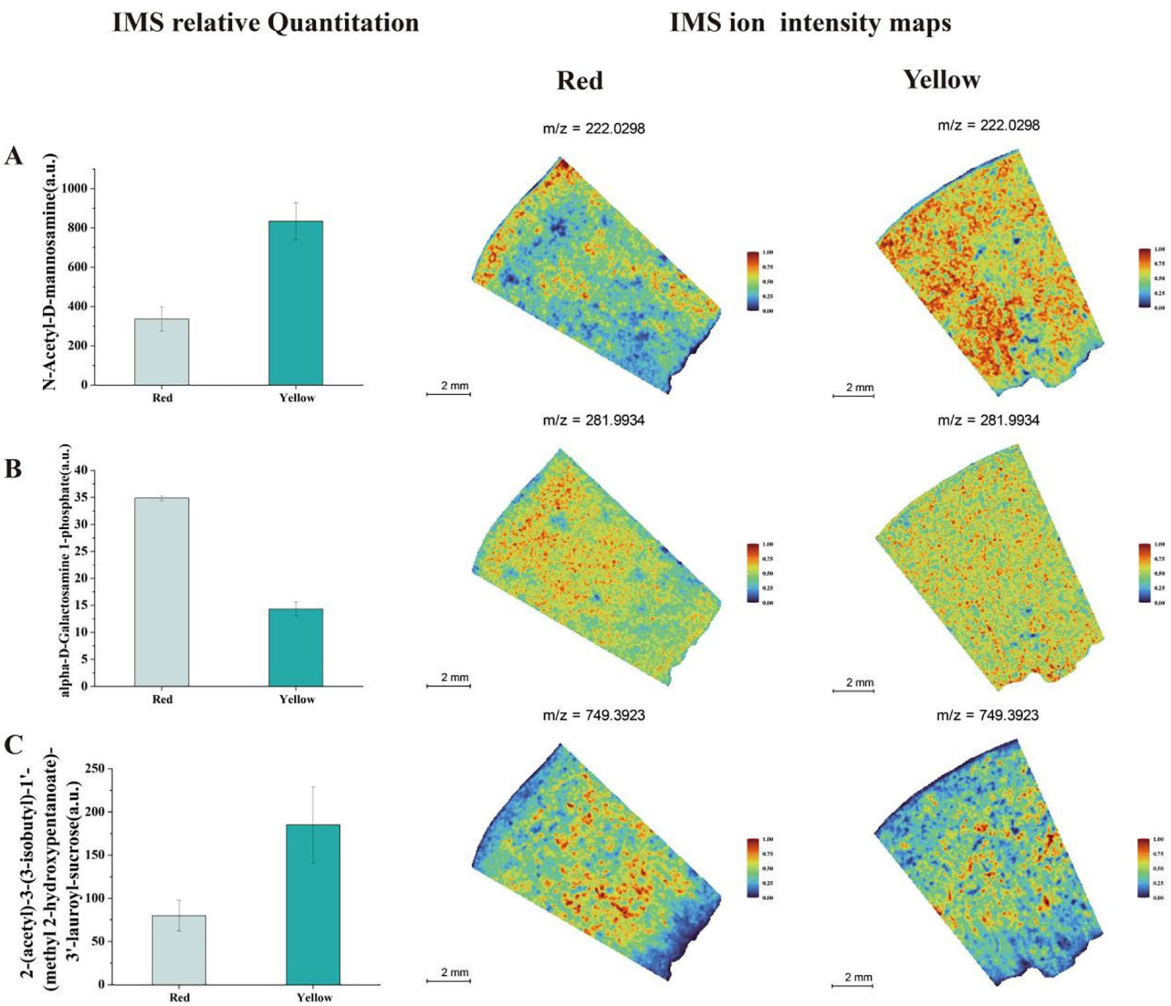
Analysis of IMS relative quantitation and IMS ion intensity maps for soluble sugar metabolites in two different color regions of apricot fruit: **(A)** N-Acetyl-D- N-Acetyl-D-mannosamine **(B)** Alpha-D-Galactosamine 1-phosphate **(C)** 2-(Acetyl)-3-(3-isobutyl)-1’-(methyl 2-hydroxypentanoate)-3’-lauroyl-sucrose.

By integrating physiological measurements with spatial metabolomics data, we identified significant spatial variations in sugar distribution between different colored fruit tissues. As shown in **Figure 4A**, the signal intensity of N-Acetyl-D-mannosamine was relatively low in the red fruit flesh. This metabolite exhibited a localized distribution in the red region but showed a stronger signal near the peel, indicating that sugar biosynthesis is more active in the peel region. In contrast, the yellow fruit exhibited a significantly stronger N-Acetyl-D-mannosamine signal, which was more evenly distributed across the peel, central fruit flesh, and areas near the seed, suggesting a more uniform sugar distribution (**Figure 4B**). Additionally, Alpha-D-Galactosamine 1-phosphate Inhibitor showed a strong signal in the red fruit flesh, primarily concentrated in the region between the peel and inner flesh. This finding suggests that the red fruit region exhibits higher glucosidase activity, leading to more active sugar hydrolysis and conversion. In contrast, the yellow fruit showed weaker and more uniform distribution of this metabolite, with particularly low concentrations near the seed. Another metabolite, 2-(Acetyl)-3-(3-isobutyl)-1’-(methyl 2-hydroxypentanoate)-3’-lauroyl-sucrose, exhibited high concentrations in both red and yellow fruit regions (**Figure 4C**). However, its abundance was relatively lower in areas adjacent to the peel and seed, indicating spatially distinct metabolic activity in different fruit regions. These findings highlight the complex spatial heterogeneity of sugar metabolism in apricot fruit, with differences in metabolite distribution reflecting localized metabolic regulation, sugar biosynthesis activity, and enzymatic conversion processes in different tissue regions.

**Figure 5:**
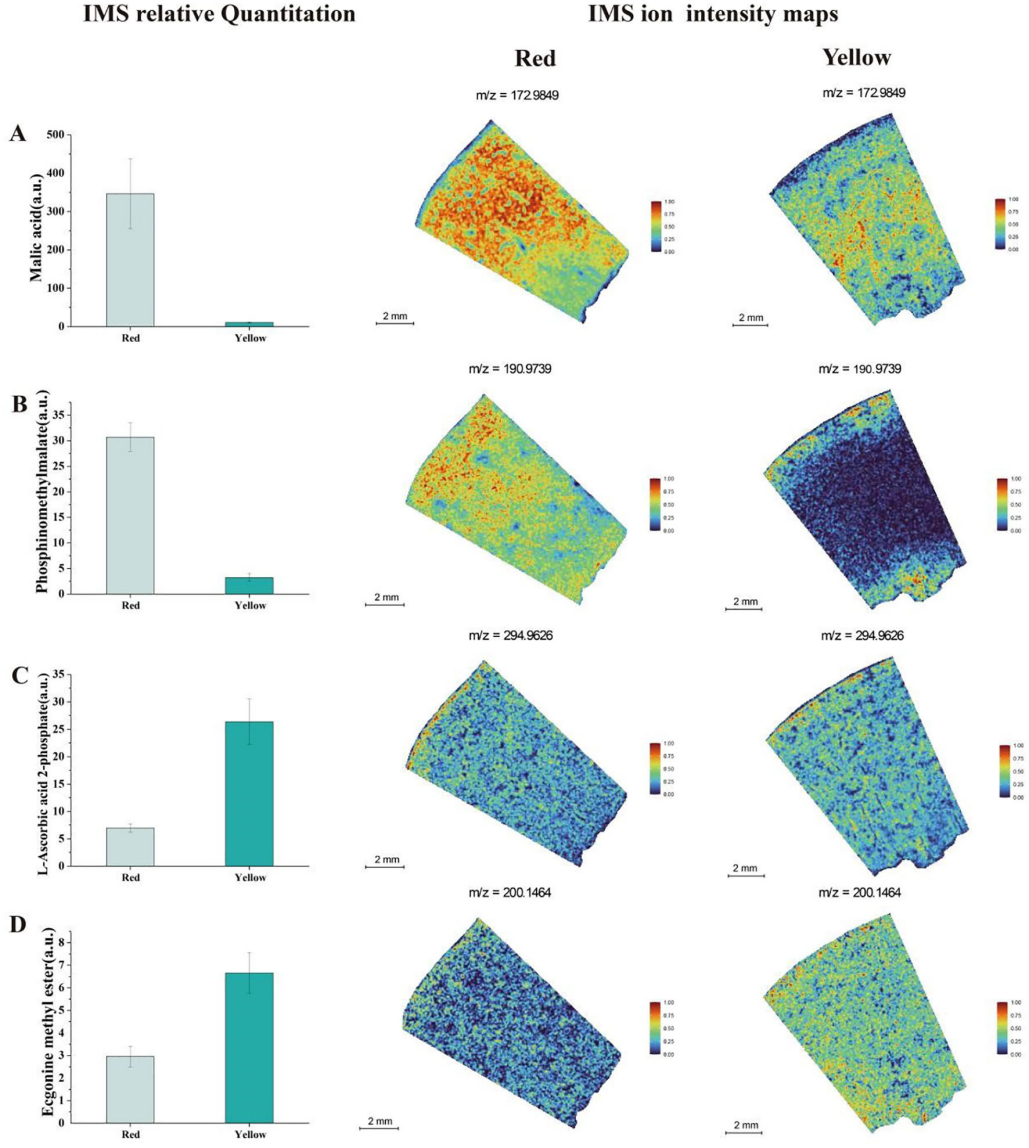
Analysis of IMS relative quantitation and IMS ion intensity maps for titratable acid metabolites in two different color regions of apricot fruit: **(A)** Malic Acid **(B)** Phosphinomethyl Malate **(C)** L-Ascorbic Acid 2-Phosphate **(D)** Ecgonine Methyl Ester.

In the analysis of organic acids in apricot fruit (**Figure 5**), we found that malic acid abundances were higher in the red fruit region and were predominantly distributed in both the peel and flesh. In contrast, in the yellow fruit, malic acid was less abundant and showed a limited distribution in the central flesh. Phosphinomethyl malate was also more abundant in the red fruit region, whereas its concentration was significantly lower in the yellow fruit, where it was primarily localized in the peel and near the seed. In the yellow fruit, *L*-Ascorbic acid 2-phosphate was more abundant and widely distributed, with approximately six times the concentration found in the red fruit. However, its spatial distribution pattern remained similar in both fruit regions.

Ecgonine methyl ester was found at higher concentrations in the yellow fruit. IMS relative quantitation analysis revealed that this metabolite exhibited a relatively uniform distribution across both fruit color regions. These findings suggest that the spatial distribution of organic acids in apricot fruit is highly variable, with distinct accumulation patterns between red and yellow regions, potentially influencing fruit acidity, antioxidant capacity, and metabolic interactions in different tissue compartments.

**Figure 6:**
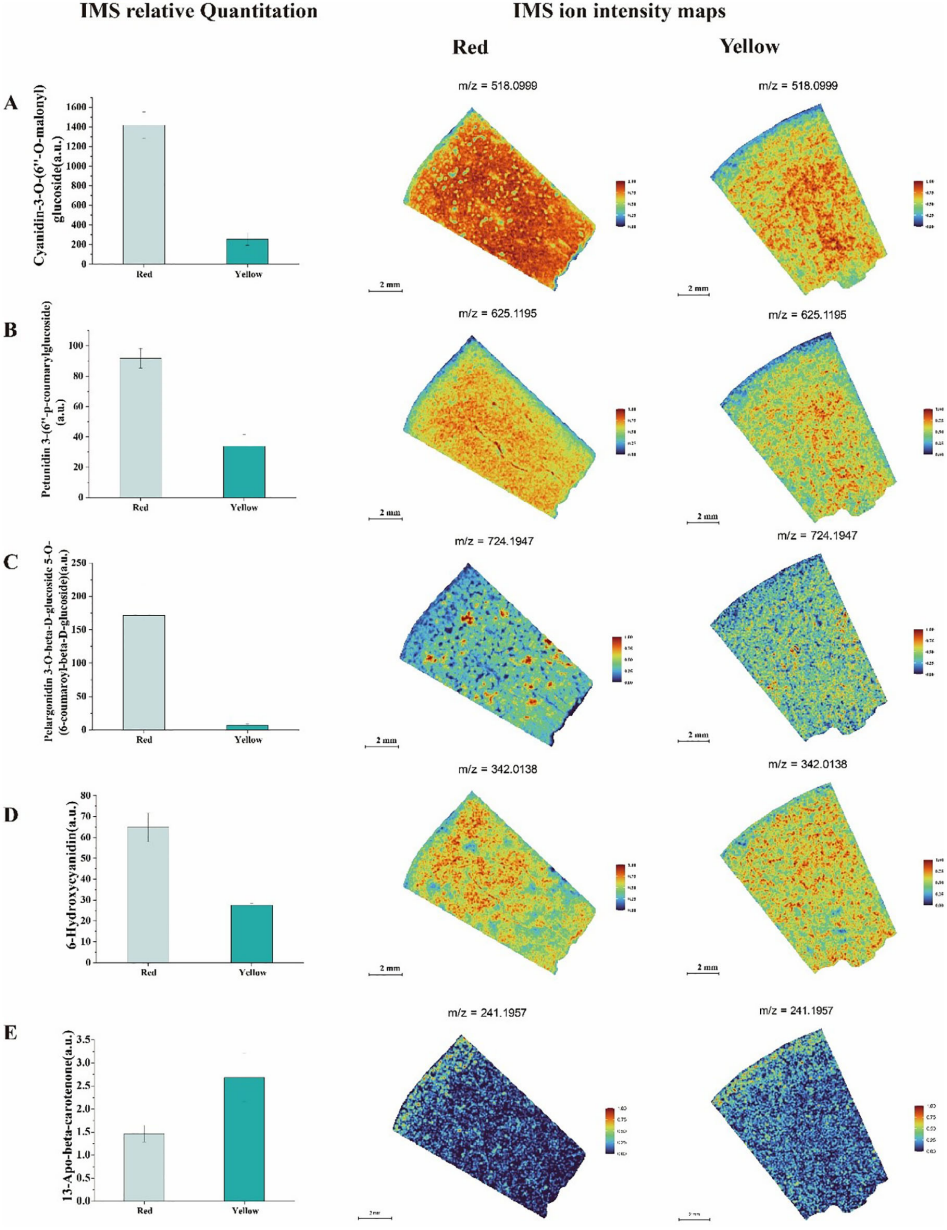
Analysis of IMS relative quantitation and IMS ion intensity maps for titratable acid metabolites in two different color regions of apricot fruit: **(A)** Cyanidin-3-O-(6’’-O-malonyl) glucoside **(B)** Petunidin 3-(6’’-p-coumarylglucoside) **(C)** Pelargonidin 3-O-beta-D-glucoside 5-O-(6-coumaroyl-beta-D-glucoside) **(D)** 6-Hydroxycyanidin **(E)** 13-Apo-beta-carotenone.

Our study revealed significant differences in pigment accumulation (**Figure 6**), antioxidant capacity, and flavor formation between the red and yellow fruit regions. The red fruit flesh was rich in various anthocyanins, including Cyanidin-3-O-(6’’-O-malonyl) glucoside, Petunidin 3-(6’’-p-coumarylglucoside), and Pelargonidin 3-O-beta-D-glucoside 5-O-(6-coumaroyl-beta-D-glucoside). These compounds not only enhanced pigment deposition, resulting in a more vibrant red coloration, but also conferred stronger antioxidant properties. IMS relative quantitation analysis indicated that these anthocyanins were predominantly distributed in the peel and adjacent fruit flesh of the red region, suggesting a crucial role in peel protection and pigment biosynthesis. In contrast, the yellow fruit flesh exhibited lower anthocyanin abundances, leading to a lighter coloration. Petunidin 3-(6’’-p-coumarylglucoside) was more abundant in the red fruit and was evenly distributed throughout the entire tissue section.

However, in the yellow fruit (**Figure 6**), its abundances was lower and primarily localized in the fruit flesh and near the seed. Similarly, Pelargonidin 3-O-beta-D-glucoside 5-O-(6-coumaroyl-beta-D-glucoside) accumulated at higher levels in the red fruit, with IMS relative quantitation showing its presence in both the flesh and seed regions. In contrast, its abundances in the yellow fruit was lower and exhibited a more uniform distribution. Additionally, 6-Hydroxycyanidin was found at higher abundances levels in the red fruit, with heatmap analysis revealing its predominant localization in the peel and flesh regions. In the yellow fruit, this compound was more evenly distributed but with an overall lower abundances. Notably, 13-Apo-beta-carotenone, a key carotenoid derivative, was significantly more abundant in the yellow fruit compared to the red fruit. Heatmap visualization indicated that its signal was concentrated in the peel and flesh regions, suggesting that the yellow fruit had a more active carotenoid accumulation pathway. This pattern of distribution implies that carotenoid biosynthesis in the yellow fruit may contribute not only to its coloration but also to its enhanced sweetness.

## 4 Discussion

This study examined the metabolic differences in sugar-acid metabolism and pigment accumulation between the red and yellow regions of *Yanzhihong* apricot fruit. The findings revealed significant variations in the content of sugars, ascorbic acid, organic acids, and carotenoids between the two color regions. These differences suggest that the two fruit regions may adopt distinct metabolic strategies during maturation, influencing fruit flavor and coloration through variations in metabolic pathways and enzyme activities.

The coloration of apricot fruit is determined by the combined effects of multiple pigments (Ruiz et al., 2005), with anthocyanins and carotenoids being the primary contributors. Anthocyanins are naturally occurring phenolic compounds responsible for the red and purple hues observed in flowers, fruits, and leaves (Kan and Bostan, 2010b). This study confirmed that anthocyanin levels were significantly higher in the red fruit region than in the yellow region, consistent with previous research (Huang et al., 2019). Additionally, carotenoid content is typically higher in orange-colored fruit (Ayour et al., 2016), and our results similarly indicated higher carotenoid accumulation in the red region compared to the yellow region. However, a significant negative correlation was observed between anthocyanins and carotenoids, indicating potential competition in their biosynthetic pathways. This phenomenon could be further validated through transcriptomic or enzyme activity analyses.

Previous studies have shown that red fruit regions, due to their high anthocyanin content, exhibit stronger antioxidant capacity, with the peel demonstrating higher antioxidant potential than the flesh, and the overall antioxidant capacity of red fruit surpassing that of yellow fruit (Zhao et al., 2024). This findings further identified a high concentration of anthocyanins, including Cyanidin-3-O-(6’’-O-malonyl)glucoside, Petunidin 3-(6’’-p-coumarylglucoside), and Pelargonidin 3-O-beta-D-glucoside 5-O-(6-coumaroyl-beta-D-glucoside), in the red fruit flesh. These compounds not only enhance pigment deposition, giving the red fruit its vivid coloration, but also contribute to its strong antioxidant capacity (Crecente-Campo et al., 2012). IMS relative quantitation revealed that these anthocyanins were primarily localized in the peel and adjacent flesh, consistent with findings in blueberry fruit, where anthocyanins are widely distributed in both the peel and flesh (Yoshimura et al., 2012), further supporting their key role in pigmentation and protective mechanisms. Notably, 13-Apo-beta-carotenone, a key carotenoid derivative, was significantly more abundant in the yellow fruit region than in the red region. IMS analysis showed that this compound was concentrated in both the peel and flesh. Carotenoids not only contribute to fruit flavor but also enhance sweetness and the foemation of aromatic compound. The more active carotenoid accumulation in the yellow fruit may be associated with its sweetness and aroma characteristics (Dragovic-Uzelac et al., 2007). Additionally, red fruit regions, which are typically rich in anthocyanins, may inhibit chlorophyll degradation, leading to relatively higher chlorophyll content in the red fruit (Zong et al., 2023). The accumulation of anthocyanins is regulated by multiple factors, including sugar levels, light exposure, temperature, and plant hormones. However, in this study, no clear relationship between anthocyanins and soluble sugars was observed, which may be due to the differential effects of sugars on anthocyanin biosynthesis across fruit types.

Our results also revealed significant differences in the distribution of organic acids between the red and yellow fruit regions, particularly ascorbic acid, malic acid, and citric acid. The yellow fruit exhibited higher levels of these organic acids than the red fruit. Ascorbic acid, a potent antioxidant, showed a strong positive correlation with citric acid, suggesting that the accumulation of these organic acids in the yellow fruit may enhance its antioxidant potential during ripening (Crecente-Campo et al., 2012; Liao et al., 2023). In terms of spatial distribution, Ecgonine methyl ester was found to be uniformly distributed in both fruit regions at relatively high concentrations, influencing overall fruit flavor. These findings suggest that citric acid is the dominant acid in the mature *Yanzhihong* apricot fruit. High levels of ascorbic acid not only mitigate oxidative stress in fruit but also enhance antioxidant capacity and promote the accumulation of sucrose and carotenoids (Davey et al., 2000; Mellidou and Kanellis, 2017; Fenech et al., 2019). Sucrose is considered a key determinant of fruit sweetness (Ackermann et al., 1992; Kroger et al., 2006), and previous studies have shown that sucrose accumulation is closely linked to sweetness formation in red fruit (Hrazdina et al., 1984; Seyffert, 1987; Boss et al., 1996; Das et al., 2012; Zhang et al., 2013; Li et al., 2014; Liu et al., 2024); however, no significant correlation was observed in this study. Moreover, there are limited reports on the relationship between soluble sugars and anthocyanins in apricot fruit. The accumulation of sugars and pigments is regulated by multiple factors, such as sugar synthesis and degradation, as well as environmental influences like light exposure and temperature, which may affect enzyme activity in metabolic pathways, ultimately influencing the sugar-acid balance and pigment accumulation.

In summary, the yellow fruit region showed a higher tendency to accumulate fructose and organic acids, resulting in a tart flavor and enhanced antioxidant potential. Conversely, the red fruit region exhibited higher levels of sucrose, carotenoids, and anthocyanins, contributing to a sweeter taste and more vibrant coloration. Despite providing valuable insights, this study has certain limitations. First, it relied solely on spatial metabolomics data without validation through transcriptomic or proteomic analyses, making it difficult to comprehensively elucidate the regulatory mechanisms underlying sugar-acid metabolism and pigment biosynthesis. Additionally, the study only examined the metabolic characteristics at the fruit maturation stage, without considering earlier developmental stages. Future research should integrate transcriptomic and proteomic data to explore metabolic regulatory mechanisms across different growth stages, providing a more comprehensive understanding of pigment and sugar-acid metabolism dynamics in *Yanzhihong* apricot fruit.

## 5 Conclusion

This study, for the first time, utilized spatial metabolomics to reveal significant spatial differences in sugar-acid metabolism and pigment accumulation across different color regions of *Yanzhihong* apricot fruit, providing a theoretical foundation for understanding the spatial regulation of fruit quality. The yellow fruit region exhibited a higher accumulation of fructose and organic acids (particularly ascorbic acid, malic acid, and citric acid), contributing to its pronounced tartness and strong antioxidant potential. In contrast, the red fruit region was characterized by higher sucrose and carotenoid levels, enhancing sweetness and promoting a more vivid coloration. Anthocyanin accumulation was significantly higher in the red fruit region, especially in the peel and flesh. The key anthocyanins identified included Cyanidin-3-O-(6’’-O-malonyl)glucoside, Petunidin 3-(6’’-p-coumarylglucoside), and Pelargonidin 3-O-beta-D-glucoside 5-O-(6-coumaroyl-beta-D-glucoside), which not only intensified the fruit’s visual appeal but also contributed to its strong antioxidant capacity. Furthermore, a significant negative correlation was observed between anthocyanins and carotenoids, suggesting their co-regulation in determining fruit coloration. The yellow fruit region exhibited higher accumulations of fructose and organic acids (citric acid, malic acid, ascorbic acid), indicating stronger sugar storage capabilities and enhanced sourness. Overall, these findings enhance our understanding of the spatial metabolic mechanisms underlying fruit quality and provide a foundation for future studies exploring the molecular regulatory pathways involved in pigment biosynthesis and sugar-acid metabolism in apricot fruit.

## Data Availability

The datasets presented in this study can be found in online repositories. The names of the repository/repositories and accession number(s) can be found in the article/**Supplementary Material**.
