# Supplementary figures and images for: Spatial metabolic heterogeneity of sugar–acid balance and pigment accumulation in distinct color regions of Yanzhihong apricot (Prunus armeniaca L.) revealed by MALDI-IMS
Source: Front Plant Sci. 2025 Aug 14;16:1636734. doi: 10.3389/fpls.2025.1636734 (PMC12391084; doi:10.3389/fpls.2025.1636734)

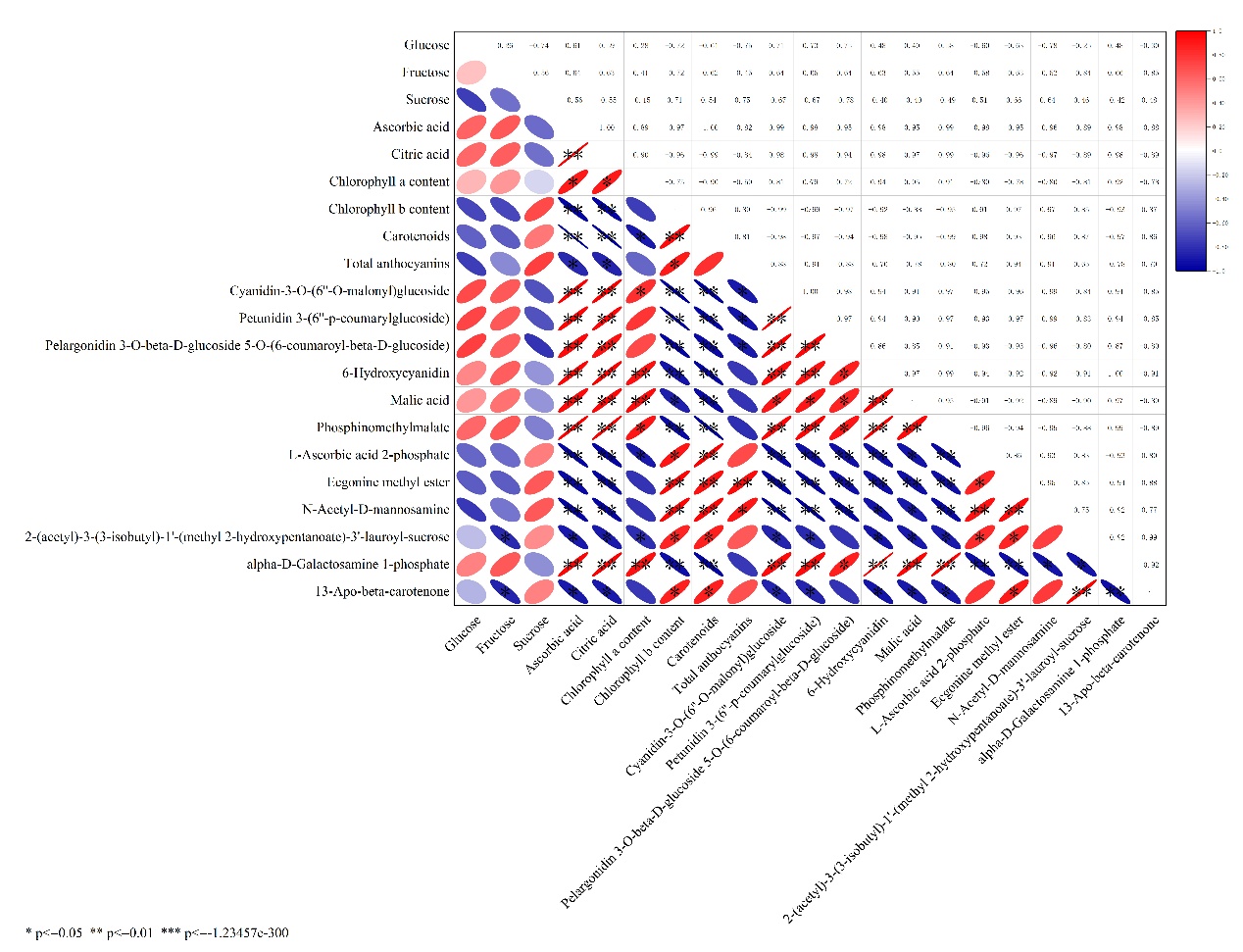


Fig. S1: relation Among Physiological Indices in Yanzhihong Apricot.

Supplement: Supplementary file 1 [file Table1.docx]
